# Special issue on heat and mass transfer in frost and ice

**DOI:** 10.1098/rsta.2024.0370

**Published:** 2025-07-17

**Authors:** S. A. Sherif, Nesrin Ozalp

**Affiliations:** ^1^ Mechanical and Aerospace Engineering, University of Florida, Gainesville, FL, USA; ^2^ Mechanical Engineering, Illinois State University, Normal, IL 61790, USA

**Keywords:** frost, ice, atmospheric environment, freezers

The physics of frost and ice is complex. They both form under a variety of conditions and deposit on surfaces of different components and systems in a myriad of application areas that affect our lives. For example, in refrigeration and air-conditioning applications, the frost formation problem has been of interest to researchers for over a century. In applications involving the preservation of food under sub-freezing temperatures, the frost formation problem is a relevant one and can have significant economic ramifications if not properly addressed. This is especially true in large installations such as those found in supermarkets and food distribution warehouses. In air-conditioning applications, especially those involving the use of heat pumps in colder climates for heating purposes, outdoor coils could get frosted up and cause a degradation in the heat-transfer performance of the outdoor coil and consequently a degradation in the performance of the entire heat-pump heating system. In the aviation industry, ice accretion on aircraft wings can pose a significant flight safety risk, as it affects the lift of the entire aircraft as well as the accuracy of the different sensors that are placed on the outside of the aircraft body to monitor the speed and other relevant flight data. False sensor readings due to ice accumulation can trigger a series of wrong steps that could cause the aircraft to lose elevation and result in accidents. Aircraft icing was of interest to researchers well before World War II, especially in military applications. Much of the literature on aircraft icing in the 1940s and the 1950s in the United States could be found in technical reports and technical memoranda belonging to the National Advisory Committee for Aeronautics (NACA) and to the US Air Force. After the National Aeronautics and Space Administration was established on 29 July 1958, subsequent aircraft icing research continued at an elevated pace.

In recognition of the importance of frost and ice in our lives, we present this theme issue where world-class experts in frost and ice are invited to address state-of-the-art issues in these two areas. The theme issue is organized with this Preface laying the framework of the different challenges encountered. The four papers that follow the Preface are all focused on ice accretion on aircraft wings. They address topics such as an improved Messinger/Myers model in unsteady aircraft icing with variable properties, a model for a Stokes-dependent droplet collection efficiency on a NACA 0012 airfoil from droplet-informed simulations with statistical overloading and using the iceAccretionFoam solver to model both rime ice (3rd paper) and glaze ice (4th paper). The 5th paper is a review of the recent advances in modelling frost formation in mechanical systems, while the 6th paper is a review of frost formation utilizing the electrohydrodynamic technique. The 7th paper presents a high-fidelity numerical simulation of frost formation and heat transfer on fin-and-tube heat exchangers in the turbulent cross-flow of humid air. The 8th paper examines the effects of the growth and freezing of condensate droplets on the initial frost thickness and density of sub-freezing surfaces. The 9th paper discusses frosting on porous membranes in energy exchangers, which are devices that transfer thermal energy between two or more fluids of different temperatures without mixing them. These energy exchangers are typically used in various industries to optimize energy use. The 10th paper presents experimental and theoretical analyses of the cooling and freezing of droplets in contact with a cold substrate with a special emphasis on the effect of the wettability of the substrate. Finally, the 11th paper explores the use of surface treatments such as surface micropatterning to promote frost-layer delamination as a technique to improve the effectiveness of the defrosting process.

The guest editors are grateful to all authors and reviewers for contributing to making this theme issue a success. The guest editors are gratefully indebted to the Editor-in-Chief of *Philosophical Transactions of the Royal Society A* for accepting our proposal to publish this theme issue. The guest editors are especially grateful to the past commissioning editor Ms. Alice Power and to the current commissioning editor Ms. Ellen Porter for their hard work and dedication and for the excellent guidance they have been providing throughout the whole process. The guest editors are equally grateful to Ms. Helen Eaton who acted as an interim commissioning editor during the transition time from Alice Power to Ellen Porter. All three commissioning editors have been nothing short of awesome.

Guest Editors:

Dr. S.A. Sherif

Professor and MAE Excellence Term Professor

Department of Mechanical and Aerospace Engineering

University of Florida

1064 Center Drive, 181 NEB Building

Gainesville, FL 32611, USA

sasherif@ufl.edu

Dr. Nesrin Ozalp

Professor and Chair

Department of Mechanical Engineering

Illinois State University

OU Old Union 301b

Campus Box 6000

Normal, IL 61790-6000, USA

nozalp@ilstu.edu

## Data Availability

This article has no additional data.

